# Percivall Pott (1713-1788): Father of Orthopaedics and Pioneer of Occupational Medicine

**DOI:** 10.7759/cureus.70608

**Published:** 2024-10-01

**Authors:** Seth Forster

**Affiliations:** 1 Trauma and Orthopaedics, Royal Shrewsbury Hospital, Shrewsbury, GBR

**Keywords:** biography, historical vignette, occupational medicine, orthopaedics, urologic cancer

## Abstract

Percivall Pott was an eighteenth-century English surgeon best known for three eponymous diseases: Pott’s fracture, Pott’s puffy tumour and Pott’s disease of the spine. He wrote extensively, with treatises covering a wide range of surgical subjects, including cataracts, cranial trauma, hernias and neurology. Pott's practice came at a time when surgery was being transformed from the work of barbers into a scientific study. His publications had a wide-reaching impact, influencing contemporary practice and setting out building blocks for the work of those who came after him.

This article aims to explore the life and career of Percivall Pott, looking in particular at his influence on the subjects of orthopaedics and occupational medicine.

## Introduction and background

**Figure 1:**
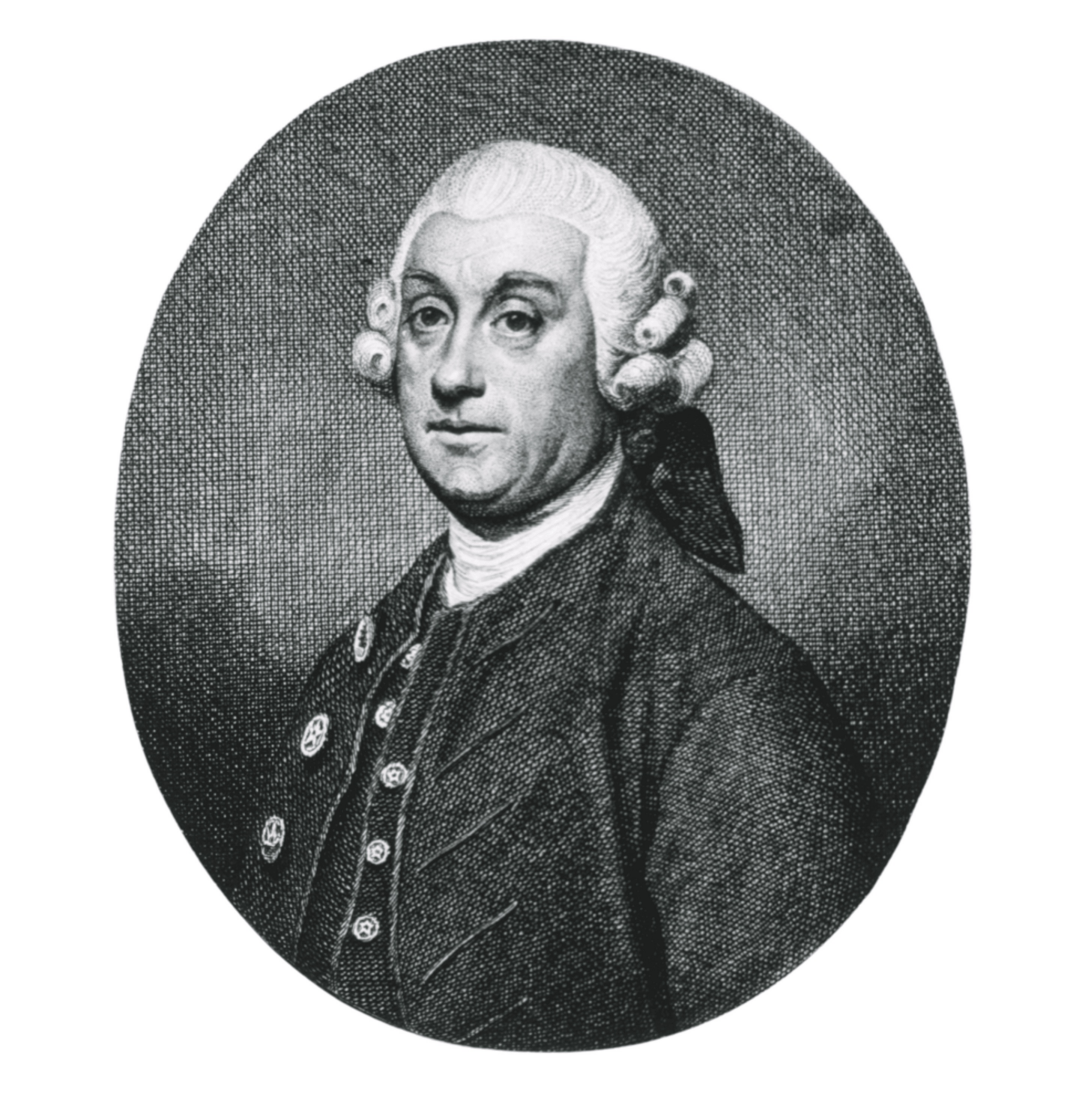
Percivall Pott F.R.S. Published by Hedges E. 1775. London. Image obtained from the National Library of Medicine, published under Creative Commons 1.0 - Public Domain [1]

Percivall Pott (Figure 1) worked in St Bartholomew’s hospital in London "man and boy, for about fifty years" [1,2]. He worked in a time of rapid change in the surgical community, as the profession transformed from the work of barbers and local healers towards a scientific discipline. Pott was one of the first members of the Company of Surgeons, a predecessor of the modern Royal College of Surgeons as it split apart from the Barber-Surgeon company in 1745. He is considered by some to be "the earliest surgeon of the modern type" [2].

Pott published 40 monographs over his career, describing his experiences with a wide range of subjects [3]. A keen tutor and lecturer, he helped to develop the next generation of English surgeons. Sir D’Arcy Power describes his legacy as "he made plain the paths so that his followers walked along them more easily and were able to go further" [4].

After a brief biography of his life and career, this article aims to describe his impact on two fields in particular. The first was orthopaedics following his treatise on "Fractures and Dislocations", and the second was his pioneering work in occupational medicine with a description of the link between scrotal cancer and chimney sweeps, which was the first description of an occupational hazard-causing cancer [5].

## Review

Life and career

Pott was born in London in 1713, the son of a scribe and notary. Four years later, Pott’s father died, leaving the family in financial difficulty [4]. He was raised by his mother with the help of a distant relative, Dr Joseph Wilcox, the Bishop of Gloucester and Rochester, who paid for Pott’s education. Pott was destined for a career in the church but at the age of 15, he changed direction and applied for an apprenticeship with a surgeon [3,6].

For the fee of 200 guineas, he became an apprentice to Edward Nourse (1701-1761), an assistant surgeon at St Bartholomew’s Hospital in London. Mr Nourse gave lectures in anatomy, and one of Pott’s tasks as his apprentice was to carry out the prosections during these lectures on cadavers. This was where he learnt the principles of medicine and anatomy, along with developing his surgical skills [7].

After an apprenticeship of seven years, he passed his exams and entered The Company of Barber-Surgeons. He initially worked in Fenchurch Street and then returned to St Bartholomew’s Hospital as an assistant surgeon when his mentor Edward Nourse became a full surgeon at the hospital [8]. Pott became a full surgeon at the hospital in 1749 and continued to work there until his retirement 38 years later [6].

In 1756, Pott had a major accident where he was thrown from his horse and sustained an open fracture of his leg. Feeling that he could not be a good clinical judge in his own case, he was carried to see other surgeons to decide on his treatment. As was common practice at the time, amputation was recommended. Whilst the surgeons were preparing for the operation, Nourse arrived to give his opinion. He convinced them to try reduction, using traction and splinting to correct the bony misalignment. This treatment was successful but left Pott bed-bound for three months [2-4,6,7].

Pott used this time to start writing, publishing his first major work "A Treatise on Ruptures" (1756), which looked at the management of hernias [4]. Following its success, he wrote 40 monographs, covering subjects from ophthalmology, orthopaedics, neurology and many others [7]. It is from these publications that the eponymous disease names for which he is best known today have originated.

Firstly, "Potts fracture", which is commonly misused to describe all ankle fractures, refers specifically to a lower fibula fracture with medial ligament displacement and tibial dislocation and was described by Pott in his 1758 monograph [7]. It is often confused as being named after the injury he sustained while horse riding, which was actually a tibial fracture distinct from ankle injuries [3].

Secondly, "Pott’s puffy tumour" is a peri-osteal abscess caused by osteomyelitis of the skull leading to forehead swelling. It may be caused by severe sinusitis, head trauma or other inflammatory conditions [6].

Finally, "Pott’s disease" of the spine is the condition for which he is best known today. This is the destruction of spinal vertebrae due to infection from tuberculosis. Progression of the disease can result in spinal curvature and compression of the spinal cord, causing nerve palsy [3,6].

As well as his written treatises, Pott was a keen teacher of the next generation of surgeons. He held large classes at home and his pupils included noted future surgeons such as James Earl (1755-1817), John Hunter (1728-1793) and John Abernathy (1764-1831) [4]. He was known for his patient-centred approach to operating, writing "in performing an operation, always remember you have a diseased body… give the least pain imaginable, be not too quick to strive to show your dexterity" [7]. This was an uncommon attribute at a time when surgical skill was often measured by speed of operation.

In 1746, he married Sarah Cruttenden and had nine children, five boys and four girls. One of his daughters married his former pupil, surgeon James Earl [4]. In 1787, he retired from operating at the age of 73 after injuring his hand, finishing a 38-year career as a lead surgeon [4,6].

Just one year later, in 1788, Percivall Pott died after a short battle with pneumonia. During his illness, he reflected on his life's work saying, "my lamp is almost extinguished, I hope it has burned for the benefit of others" [2].

Progressive work in orthopaedics

Pott is considered by some to be one of the founders of orthopaedics as a surgical specialty [3]. At the start of the 1700s, broken bones were dealt with by local bone setters or farriers. It was not felt to be a complicated practice and as such was done by people with very little training or experience; Pott wrote "every, the most inexpert and least instructed practioners deems himself perfectly qualified to fulfil this part of the chirurgic craft" [9].

He wrote his 126-page monograph "Some Few General Remarks on Fractures and Dislocations" in 1758, saying "It is by no means my intention to write a regular treatise on fractures, I mean only to throw out a few hints" [7]. Understated though it may be, the work was considered one of his most important publications. It set out principles of practice, changing the contemporary amateur methods of treatment and suggesting alternative methods that were then practised until well into the nineteenth century [2,7].

Standard practice for long bone fractures at the time was to treat them with strict limb extension. Pott wrote that this practice led to muscle tension, future deformity and lameness. Using known examples of humeral fractures being treated with the elbow flexed and placed in a sling, he suggested using flexion on other long bones to reduce the tension on the muscles that displaced healing fractures [9]. He used bed rest, positioning patients on their sides and flexing their knees and feet to treat femoral and tibial fractures [7]. His methods were significantly more effective than previous practice and were taken up by other eighteenth-century surgeons [4].

He is best known in orthopaedics for his work on ankle fractures. As the first person to create a classification system for these injuries, he described them depending on the number of malleoli involved, either uni- or bimalleolar. He wrote about the importance of the fibula in maintaining alignment and stability of the ankle, despite its small size compared to the tibia [4,9]. Pott drew particular attention to a lower fibula fracture with medial ligament disruption and tibial dislocation, which thenceforth became known as Pott’s fracture. His suggested treatment of side posture rest on pillows with a flexed knee to reduce the dislocation led to lower future incidences of deformity and disability [7].

Pioneering work in occupational medicine

In his 1775 paper "Chirurgical Observations" Pott described a link between chimney sweeps and the risk of developing scrotal cancer [3]. Pott was not the first to write about scrotal cancer, nor to discuss occupational diseases, which had been known since ancient times in the context of lead workers. His paper was, however, the first-known report of an occupational hazard causing cancer and as such, was instrumental in the foundation of occupational medicine as a subject [10].

He wrote:

"There is a disease as peculiar to a certain set of people, which has not, at least to my knowledge been publicly noticed; I mean the chimney sweeps cancer. It is a disease which always makes its first attack on, and first appearance in the inferior part of the scrotum; where it produced a superficial, painful, ragged, ill-looking sore, with hard and rising edges: The trade call it soot wart" [11].

In 1700s London, chimneys for fireplaces were narrow, twisted and irregular. Soot would build up on the inside of these and would have to be removed by hand, as it was a fire hazard. Due to the construction of these chimneys, the only people who could access these soot deposits were small boys, often aged between four and seven who would climb up the chimneys, using their hands and knees, sometimes naked to fit into smaller spaces [5].

**Figure 2:**
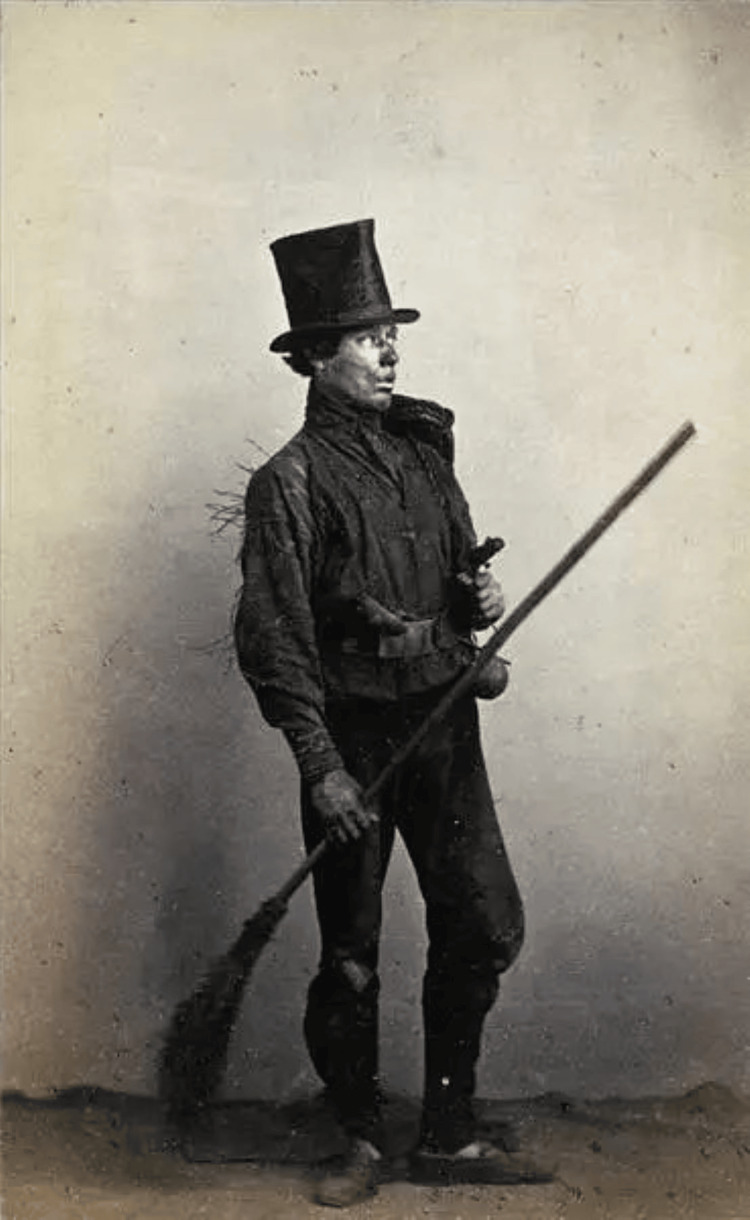
Photograph of an older chimney sweep from the 1800s Image obtained from ww.owlcation.com. Image published under Creative Commons 1.0 - Public Domain [12].

These chimney sweeps (Figure 2) worked in appalling conditions, risking suffocation, heat from recent fires and entrapment in the chimneys before facing a future of scrotal cancer. Pott remarked that although others could also develop scrotal cancers, it was a disease that sweeps were particularly liable to [11]. Between 1880 and 1890, St Bartholomew’s Hospital treated 39 cases of scrotal cancer, 29 of which were in sweeps [6].

For treatment, Pott recommended early surgery as the best option. He suggested the cause of the disease in this population was the lodgement of soot amongst the rugae of the scrotum [11]. This cause was widely debated, with others suggesting it was friction-related. It wasn’t until Passey in 1922 was able to induce skin cancer in mice with an extract from soot that the cause was confirmed [10]. The advice usually given for prevention was regular washing and changing of clothes [6].

His publication was soon followed by reports from other surgeons, and public opinion began moving against the use of young children as chimney sweeps [5]. His work helped to improve conditions for sweeps, and in 1788, the first British child labour law "The Chimney Sweepers Act" was adopted, mandating that children under eight could not climb chimneys and their employers must wash them at least once a week [3].

The act was poorly enforced. Efforts to restrict the use of child chimney sweepers were opposed in parliament, mostly due to insurance companies who believed that mechanical alternatives were less effective than children [10]. There were further attempts at regulating the practice with parliamentary acts in 1834, 1840, and 1864. These all also had limited success. A final act in 1875 chaired by Lord Salisbury was successful in imposing a system of licensing on chimney sweep masters (who employed the children), ensuring no-one under 21 could climb a chimney and making the police responsible for upholding all the previous acts [10].

Scrotal cancer is now a disease of comparative rarity. This is due, in part, to legislation against the use of climbing boys and the improvement of mechanical devices to clean chimneys instead [13]. In his publication "Chirurgical Observations", Pott not only helped found the study of occupational oncology but also had far-reaching influences on the use of child labour.

## Conclusions

Percivall Pott was at the forefront of improvements in surgical practice during the 1700s. His teaching and publications helped to turn surgery into the scientific, evidence-based discipline it is today. Whilst best remembered for his three eponymous disease names, his advances in a range of different specialties and his societal impact give him a far wider-reaching legacy than is often acknowledged.
